# Measurement of HbA1c in multicentre diabetes trials – should blood samples be tested locally or sent to a central laboratory: an agreement analysis

**DOI:** 10.1186/s13063-016-1640-6

**Published:** 2016-10-24

**Authors:** Barbara N. Arch, Joanne Blair, Andrew McKay, John W. Gregory, Paul Newland, Carrol Gamble

**Affiliations:** 1 Department of Biostatistics, The University of Liverpool, Liverpool, L69 3BX UK; 2 Alder Hey Children’s NHS FT, East Prescott Road, Liverpool, L12 2AP UK; 3 Professor in Paediatric Endocrinology & Honorary Consultant, Division of Population Medicine, School of Medicine, Cardiff University, Heath Park, Cardiff, CF14 4XN UK; 4 Department of Biochemistry, Alder Hey Children’s NHS FT, East Prescott Road, Liverpool, L122AP UK

**Keywords:** HbA1c, Agreement, Trial design, Measurement

## Abstract

**Background:**

Glycated haemoglobin (HbA1c) is an important outcome measure in diabetes clinical trials. For multicentre designs, HbA1c can be measured locally at participating centres or by sending blood samples to a central laboratory. This study analyses the agreement between local and central measurements, using 1-year follow-up data collected in a multicentre randomised controlled trial (RCT) of newly diagnosed children with type I diabetes.

**Methods:**

HbA1c measurements were routinely analysed both locally and centrally at baseline and then at 3, 6, 9 and 12 months and the data reported in mmol/mol. Agreement was assessed by calculating the bias and 95 % limits of agreement, using the Bland-Altman analysis method. A predetermined benchmark for clinically acceptable margin of error between measurements was subjectively set as ±10 % for HbA1c. The percentage of pairs of measurements that were classified as clinically acceptable was calculated. Descriptive statistics were used to examine the agreement within centres. Treatment group was not considered.

**Results:**

Five hundred and ninety pairs of measurement, representing 255 children and 15 trial centres across four follow-up time points, were compared. There was no significant bias: local measurements were an average of 0.16 mmol/mol (SD = 4.5, 95 % CI −0.2 to 0.5) higher than central. The 95 % limits of agreement were −8.6 to 9.0 mmol/mol (local minus central). Eighty percent of local measurements were within ±10 % of corresponding central measurements. Some trial centres were more varied in the differences observed between local and central measurements: IQRs ranging from 3 to 9 mmol/mol; none indicated systematic bias.

**Conclusions:**

Variation in agreement between HbA1c measurements was greater than had been expected although no overall bias was detected and standard deviations were similar. Discrepancies were present across all participating centres. These findings have implications for the comparison of standards of clinical care between centres, the design of future multicentre RCTs and existing quality assurance processes for HbA1c measurements. We recommend that centralised HbA1c measurement is preferable in the multicentre clinical trial setting.

**Trial registration:**

Eudract No. 2010-023792-25, registered on 4 November 2010.

## Background

In 2014, approximately 3.2 million people living in the United Kingdom were diagnosed with diabetes [1–5]. Globally, 12 % of all health care expenditure is spent on diabetes care [6]. In the United Kingdom, the greatest percentage of NHS costs, and burden to patients, comes from long-term vascular complications [7].

Circulating blood glucose attaches to haemoglobin and concentrations of the resulting glycated haemoglobin, or ‘HbA1c’, reflect levels of blood glucose in the preceding 8–12 weeks. In 1993 The Diabetes Control and Complications Trial (DCCT) reported conclusive evidence that glycaemic control (GC), reported as mean HbA1c, is a critical determinant of the risk of long-term vascular complications [8]. More recently, variability of HbA1c over time has been identified as an important, additional risk factor for vascular disease. During the course of the DCCT, a patient in the 97.5th centile of HbA1c variability was three times more likely to develop diabetes-related eye disease and more than twice as likely to develop diabetes-related kidney disease that of a patient in the 2.5th centile [9].

HbA1c is measured routinely in clinical services to guide individual diabetes care. It is also a robust clinical outcome for clinical trials, being largely influenced by circulating blood glucose levels. The only caveat is that subjects with blood disorders, such as sickle cell anaemia, may have more rapid haemoglobin turnover, leading to HbA1c concentrations providing underestimates of true blood glucose control. It is usual practice to exclude these individuals from clinical trials in which HbA1c is an outcome measure, and for other estimates of blood glucose control that are independent of haemoglobin turnover to be used in routine clinical practice.

Standards of diabetes care and clinical outcomes are reported annually at a national level, and can be compared between centres through the National Diabetes Audit and the National Paediatric Diabetes Audit. There are many factors that influence GC, of which one is the quality of clinical services.

The International Federation of Clinical Chemistry and Laboratory Medicine (IFCC) has worked to standardise measurement of HbA1c at an international level [10] and HbA1c assays have been calibrated against the IFCC-standardised values since June 2009. This process of standardisation should in theory remove the need for centralised measurement of HbA1c for either clinical or research purposes. The main analytical methods used for the measurement of HbA1c include affinity chromatography, immunoassay, cation exchange chromatography, and capillary electrophoresis [11]. Due to the variety of methods available, there can be significant differences between results obtained, as observed on the national external quality assurance programmes (due in part to haemoglobin variants and sample interferences such as bilirubin and lipids).

Due to the importance of GC in determining future risk of diabetes-related complications, it is an important endpoint used within clinical trials. In the multicentre study setting, HbA1c can be measured in three ways: at point of contact (POC), e.g. on the ward or in a clinic using bedside portable instrumentation; by sending blood samples to local hospital laboratories; or by sending blood samples to a central laboratory. Measurements made at POC and local laboratories are logistically easier, less costly and represent the pragmatic reality of HbA1c measurement in routine care. However, a central laboratory provides a single standardised testing facility that may provide more consistent results for primary endpoints leading to higher validity of conclusions and allow comparison of outcomes between participating centres. However, this approach incurs higher costs in trial implementation, and may represent an additional burden for paediatric patients, in whom it may be difficult to obtain sufficient blood for dual analyses.

SCIPI is a clinical trial randomising newly diagnosed children to two methods of insulin delivery [12]: multiple daily injections or continuous pump infusion. The study, funded by the National Institutes for Health Research (NIHR) Health Technology Assessment (HTA) programme aims to compare the effect of insulin regimen on HbA1c measurements 12 months post diagnosis. The initial application to the NIHR HTA requested costs to support central laboratory analysis of HbA1c. This was to provide uniformity and standardisation of the method of measurement. However, the funding decision stated that ‘HbA1c should be measured according to the national protocol and could be done locally’. Despite arguments to support central laboratory analysis the trial was funded to support local costs only.

The Trial Management Group (TMG) expressed concerns about the limitations of this approach and so funds were eventually identified to include centralised HbA1c testing. The central laboratory began collecting blood samples 19 months after the study opened. This meant that the first participants’ results were not from the central laboratory. Furthermore, the pragmatic nature of the study meant that local results for follow-up appointments were usually obtained using portable instrumentation. Although POC devices are calibrated to the same standards as laboratory-based methods, this introduces another level of uncertainty. Also, if the sample was taken at a patient home visit then local results may have been unobtainable but a sample was sent for central analysis. The necessity for both local and central analysis was evident within the clinical trial protocol and was stressed during the training of staff collecting samples at participating sites throughout the duration of the trial and included publicity for this issue in the trial newsletter and email reminders of its importance.

This study examines the agreement between locally and centrally measured HbA1c in a multicentre trial setting. The aim of this analysis is to inform the statistical analysis plan for the final analysis of SCIPI and future trial designs. These data also give us helpful insights into the validity of comparing HbA1c values between clinical centres as a measure of quality of care.

## Methods

At the time of this agreement analysis 294 children and young people aged 7 months to 15 years, who had been newly diagnosed with type I diabetes mellitus, had been randomised in a 1:1 ratio to receive either continuous subcutaneous insulin infusion (CSII) or multiple daily injections (MDI) of insulin. Fifteen UK trial centres were involved in the study.

The first patient was randomised on 31 May 2011 and central laboratory analysis became available on 21 January 2013. The trial completed recruitment at the end of March 2015 with 294 patients randomised but some follow-up data collection was occurring at the time of this analysis (due to be completed by the end of March 2016).

HbA1c was collected prior to the start of the randomised treatment and then at follow-up appointments at 3, 6, 9 and 12 months. Data regarding the randomised allocations was not required for this analysis as this study investigated agreement independent of treatment received.

### Measurement of HbA1c

The capillary blood samples were collected from finger-pricks into small capillary tubes and were analysed in two separate locations: locally – the majority were analysed using portable instrumentation at outpatient clinics; and centrally – at the clinical pathology laboratory at Alder Hey Children’s NHS Foundation Trust, using a Siemens DCA 2000 machine. Transportation of samples to the central laboratory was by two possible modes: either through the post; or via bespoke courier systems to ensure that the samples would be received the next day.

### Quality assurance of the measurement of HbA1c

Portable instrumentation is calibrated regularly with local laboratories. All laboratories involved in the measurement of HbA1c are obliged to participate in external quality assurance schemes, ensuring that laboratories with equivalent equipment are able to produce results that are comparable to each other. For almost all HbA1c measurements (local and central), the biochemical methodology employed was immunoassay.

### Data extraction

Pairs of HbA1c measurements (one local and one central) were extracted from the SCIPI database in September 2015. Each pair of measurements was required to be from a single blood sample (samples taken on different days were excluded). Unusually large disagreements (>20 mmol/mol) were queried and, if unresolved, were reported on but excluded from the analysis to limit bias of results.

### Units of measurement of HbA1c

HbA1c measured locally was usually recorded in two units of measurement: mmol/mol and percent (percentage of total haemoglobin). HbA1c measured at the central laboratory (at Alder Hey) was recorded in mmol/mol only, according to the IFCC-aligned standards for reporting HbA1c [10]. From 1 June 2011 the clinical standard unit of measurement for HbA1c became mmol/mol. This paper reports agreement using mmol/mol.

The formula used for conversion between the two units of measurement is:

$$ HbA 1c\ \left(\%\right)=\frac{HbA 1c\ \left(\mathrm{mmol}/\mathrm{mol}\right)}{10.929}+2.15, $$

where HbA1c was only recorded in percent, the conversion formula above was used to calculate a value in mmol/mol. Where both units of measurement were present, a check was made on the accuracy of the conversion by comparing derived conversions from the formula above with actual conversions recorded in the database. Where there was a discrepancy of more than 1 mmol/mol or 0.1 % (i.e. when not accounted for due to rounding), data entry was queried. For unresolved queries, the source unit of measurement was sought and taken to be the true measurement, and the other was derived using the conversion formula. If the source unit of measurement could not be verified, the pair of measurements was excluded.

### Clinically acceptable agreement

Prior to analysis and specified within the statistical analysis plan, limits of clinically acceptable agreement were defined by the chief investigator to be that local measurements were within ±10 % mmol/mol of central measurements:

$$ 0.9\times HbA 1{c}_{Local}<HbA 1{c}_{Central}<1.1\times HbA 1{c}_{Local}. $$

Limits are justified by the potential differences in clinical outcomes that a difference of 10 % can imply – in the Diabetes Control and Complication Research Group study [8], the relationship between HbA1c and the risk of developing microvascular complications, and the rate of progression of microvascular complications, was seen as a continuum across the range of GC. This was most marked at the higher levels of HbA1c: the 10 % difference in HbA1c between 10 % and 9 % was associated with a 25 % reduction in the rate of progression of retinopathy. Within patient observations they suggest that a difference in HbA1c of 10 % is clinically significant: those patients who reduced their HbA1c by 10 % (for example, 9.0 % to 8.1 %) reduced their risk of acquiring retinopathy and nephropathy by 39 % and 25 %, respectively.

### Statistical methods

Prior to undertaking the agreement analysis, a statistical analysis plan was developed by BA and agreed by CG. Simple demographics were summarised (gender, age and social deprivation score) for both the full cohort of children randomised in SCIPI and the subset that had at least one valid pair of HbA1c measurements included in this analysis. The Bland-Altman analysis of agreement method was used to compare local and central measurements. For each pair of measurements, the difference (*D*) (local measurement minus central) and the mean (*M*) was calculated. Heteroscedasticity (variance of *D* increasing/decreasing with increasing *M*) was examined through plotting limits of agreement (LOA) graphs. One-way analysis of variance (ANOVA) was used to check whether within-subject variance of differences was significantly different to between subject variance – i.e. to check whether the repeated measures nature of the data needed to be accounted for in the agreement analysis. The percentage of pairs within clinically acceptable limits was calculated for both units of measurement using the predetermined limits described below. Descriptive statistics were used to examine the agreement within centres. Finally, the time-lag between date of collection of blood sample and date of laboratory analysis was investigated as a possible explanation for poor agreement. All analyses were implemented using SAS software version 9.3.

## Results

### Sample characteristics

**Fig. 1:**
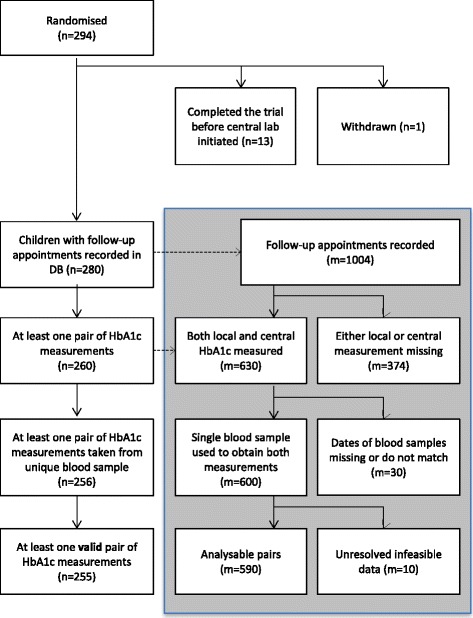
Flowchart showing sample sizes (*n*) and number of pairs of measurements (*m*) available for analysis

Of 294 children randomised for the trial, 255 had at least one pair of measurements of HbA1c included in this analysis (see below and Fig. 1 for detailed breakdown of exclusions). Of the 255 children included in this analysis, the mean (standard deviation (SD)) age was 8.9 (4.1) years and 51.6 % were male. They were similar in demography to the full sample of 294 children (aged 9 (4.1) years, 52.4 % male) (see Table 1). Children were recruited from 14 trial centres across England and from 1 centre in Wales.

**Table 1:** Sample characteristics

	All children randomised		Children with at least one valid pair of measurements	
	*N*	Summary statistics	*n*	Summary statistics
Available sample size	294		255	
Males: *n* (%)	292	153 (52.4 %)	254	131 (51.6 %)
Age: mean (SD)	292	9.0 (4.1)	254	8.9 (4.1)
Participating centres		15		15
Number of follow-up appointments attended:	294	-	255	2 (1,3)^a^
Total	294	1371	255	590^a^
Median (IQR) per child	294	5(4,5)	255	2 (1,3)^a^

^a^Time points where a valid pair of measurements were available. *IQR* interquartile range, *SD* standard deviation

A total of 590 pairs of measurements of HbA1c were included, all from follow-up: 139 at 3 months, 157 at 6 months, 143 at 9 months and 151 at 12 months. At baseline, local and central measures were taken on different days: locally (usually sent to the local laboratory) at diagnosis, and centrally at randomisation/entry into the trial. Children included in this analysis had a median of two valid pairs of measurements. Of the 15 trial centres, 6 had low sample sizes (less than 20 valid pairs) and the remaining 9 had between 21 and 108 valid pairs.

### Exclusions

There was potential for 1004 follow-up HbA1c measurements to be available at the time of the data snapshot of which 414 could not be included (see Fig. 1). Three hundred and seventy four of these were because at least one of the pair was missing (35 appointments were not attended; 35 local measurements were either not available or not measured; 65 appointments took place prior to the commencement of central laboratory testing; for 172 samples the quantity of blood sent to the central laboratory was not sufficient to enable a test; 8 samples sent to the central laboratory were clotted; and 59 were missing for some other the reason). Thirty pairs were excluded because they could not be confirmed to be from the same blood sample. In 10 pairs there were unresolved data-validity queries: 8 pairs were found to differ by more than 20 mmol/mol – there was a maximum discrepancy of 40 mmol/mol; 2 pairs had unresolved questionable unit conversions recorded.

### Bias and limits of agreement

Table 2 provides the results of HbA1c in mmol/mol at each follow-up time. The means and SDs are similar for local and central measurements both overall and by time point.

**Table 2:** Mean (SD) glycosylated haemoglobin (HbA1c) measured locally and centrally overall and by time point (valid pairs of data only)

	Number of pairs of measurements	Local (mmol/mol)	Central (mmol/mol)
All data	590	55.2 (13.0)	55.1 (13.1)
By time point:			
3 months	139	50.0 (12.9)	49.5 (12.2)
6 months	157	54.0 (12.9)	54.2 (13.5)
9 months	143	58.5 (12.5)	57.9 (12.3)
12 months	151	58.3 (11.7)	58.5 (12.3)

**Fig. 2:**
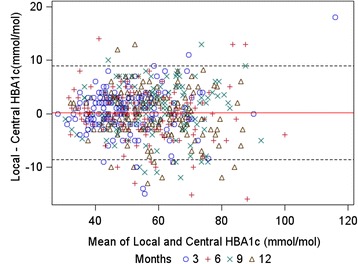
Limits of agreement (LOA) graph (95 % LOA lines: dashed, bias: red line)

On average, local measurements were 0.16 mmol/mol higher than central (95 % CI −0.2 to 0.5). This bias was not statistically significant, showing that local measurements were not systematically higher or lower than central measurements. The 95 % LOA were calculated to be −8.6 to 9.0 mmol/mol (see Fig. 2 and Table 3).

**Table 3:** Agreement statistics: local minus central glycosylated haemoglobin (HbA1c)

	HbA1c (mmol/mol)
Bias	
Mean difference	0.16
SD of differences	4.49
95 % CI for mean	(−0.20, 0.52)
95 % limits of agreement	−8.6, 9.0
Percentage within clinically acceptable limits	92.9 %

*CI* confidence interval, *SD* standard deviation

### Verification of assumptions

**Fig. 3:**
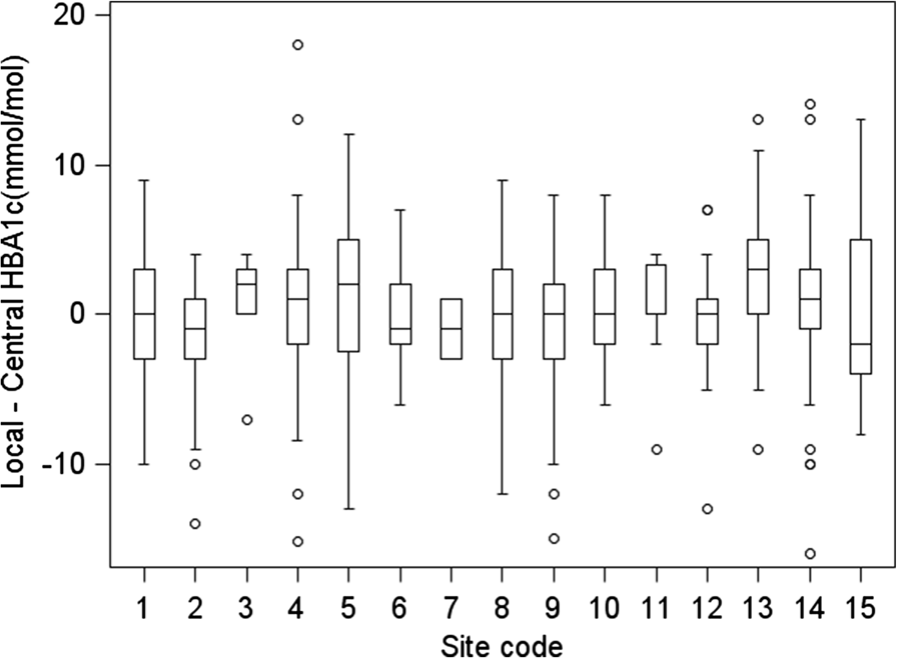
Boxplots showing the distribution of differences between local and central measurements, by trial centre

The assumptions of the Bland-Altman LOA analysis were found to hold: differences between local and central measurements were symmetrically distributed and with an approximate bell shape; and there was no indication of serious heteroscedasticity – i.e. no marked increase/decrease in variance of differences with increasing HbA1c (see Fig. 2). There was no evidence that the variance of differences was different for measurements on the same child compared with measurements on different children (one-way ANOVA *p* = 0.15) – for this reason, the repeated measures nature of the data was not taken into account in calculating LOA. Trial centre was ignored for the main agreement analysis, as the number of valid pairs of measurements for 6 out of 14 centres was small (less than 20), and the remaining 9 centres did not display strong heterogeneity in levels of agreement (Fig. 3, Table 4).

**Table 4:** Agreement by centre

Centre code	Number of children	Number of valid pairs of measurements	Median (IQR) difference (local–central) (mmol/mol)	Local within ±10 % of central (%)
1	8	13	0 (−3,3)	85 %
2	16	47	−1 (−3,1)	94 %
3	2	6	2 (0,3)	83 %
4	29	68	1 (−2,3)	78 %
5	17	28	2 (−2.5,5)	71 %
6	6	18	−1 (−2,2)	89 %
7	1	2	−1 (−3,1)	100 %
8	52	108	0 (−3,3)	79 %
9	41	94	0 (−3,2)	82 %
10	7	21	0 (−2,3)	90 %
11	7	9	0 (0,3.3)	89 %
12	10	25	0 (−2,1)	88 %
13	19	51	3 (0,5)	69 %
14	34	85	1 (−1,3)	76 %
15	6	15	−2 (−4,5)	80 %
All	255	590	0 (−2,3)	80 %

*IQR* interquartile range

### Clinically acceptable agreement

The proportion of pairs where the local measurement was within ±10 % of the central measurement was 80 % (see Table 3 for results by site).

### Centre-specific agreement

Centres varied in levels of agreement (see Table 4 and Fig. 3). Of the six centres with larger numbers of valid pairs of measurements (*m* > 30) (centres 2, 4, 8, 9, 13 and 14), one had a median difference that was negative, two had a median of 0 and three had positive medians. All six had both negative and positive differences, indicating that no centre incurred a clear systematic bias. Centre 5 was very variable with an interquartile range (IQR) for differences of 7.5 mmol/mol – 29 % of pairs from this centre were classified as clinically unacceptably different.

### Time-lags between blood collection and central analysis

There was a median time-lag of 2 days (IQR 1–4 days) between the collection of blood samples and their analysis at the central laboratory. There was no correlation (*r* = −0.02) detected between time-lag and difference between local and central measurements.

## Discussion

These data suggest that there are discrepancies in measurements of HbA1c depending on whether blood samples are analysed locally using POC equipment, or sent to a central laboratory; but that these discrepancies are not biased. It is also important to note that the SDs of measurements were similar for local and central measurements at every time point, showing that power calculations would not be affected by location of measurement. In this study setting, it was found that 95 % of local and central measurements were within a fairly wide margin of discrepancy: ±9 mmol/mol and that one in five (20 %) pairs were classified as clinically unacceptably different. This has implications for the validity and reliability of measurements for clinical decision-making for individual patients.

In the course of this study, some data entry errors, and errors in the conversion from one unit of measurement to another were found – though these were detected through the querying process. Only a handful of queried data anomalies could not be verified and these were excluded from analysis. Centre-specific agreement did vary, though there was no centre-specific systematic bias evident. Some centres may have had more unreliable local measurement methods than others or there may have been more errors with data entry.

The time-lag between blood sampling and analysis at the central laboratory was not associated with levels of agreement, suggesting that there were no issues affecting measurement related to storage and transport of blood samples.

Discrepancies between HbA1c measurements can sometimes be explained by issues with instrumentation (lot-to-lot variation with HbA1c cartridges; variation between instrumentation makes and models; differences in sample collection devices; measurement errors by users; rogue cartridges within a batch).

This study does not provide evidence that central measurements are more accurate or more reliable, but rather that the measurement of HbA1c is variable between methods of analysis. In the routine practice of diabetes care, local measurement at clinics incurs lower costs and is convenient for decision-making; in the clinical trial setting, however, a single systematic centralised methodology would be preferable to eliminate any chance that differences in measurement methodology, or levels of training, affect the estimation of effect sizes. Pragmatically, this study provides no reason why local measurements could not be used in place of missing central measurements, given that there would be no overall bias incurred.

### Conclusions

This study shows that a central laboratory provides a standardised measurement methodology for recording HbA1c during follow-up, and that the results obtained are an unbiased representation of HbA1c measured locally at trial centres. Overall, the mean and standard deviation of measurements were similar for both sources of measurement, meaning that power calculations would not be affected by choice of source of measurement. However, there was found to be a wide spread of differences between local and central measurements for individual blood samples. This means that in some cases, post-diagnosis decision-making could be quite different if based on local measurements compared with central ones.

For the purposes of developing the SCIPI analysis plan the primary analysis will use central laboratory measures where available and use local results when this is not the case. This is based on the lack of systematic bias and the consistency in the size of the means and SDs across time points and scales.

Future investigators, and those using measures of HbA1c as indicators of standards of routine clinical care need to be aware of the variability that persists in the measurement of HbA1c across centres within the United Kingdom, despite a commitment to working according to the IFCC initiative for standardisation of measuring and reporting HbA1c. A single central laboratory provides a uniform methodology that, whilst more expensive, removes some of the complexity of sources of variation inherent in the multicentre local measurement approach.

## Abbreviations

GC: Glycaemic control
HbA1c: Glycated haemoglobin
IQR: Interquartile range
LOA: Limits of agreement
POC: Point of contact
SD: Standard deviation
