# Erratum to: Measurement of HbA1c in multicentre diabetes trials – should blood samples be tested locally or sent to a central laboratory: an agreement analysis

**DOI:** 10.1186/s13063-017-1914-7

**Published:** 2017-04-05

**Authors:** Barbara N. Arch, Joanne Blair, Andrew McKay, John W. Gregory, Paul Newland, Carrol Gamble

**Affiliations:** 1 Department of Biostatistics, The University of Liverpool, Liverpool, L69 3BX UK; 2 Alder Hey Children’s NHS FT, East Prescott Road, Liverpool, L12 2AP UK; 3 Professor in Paediatric Endocrinology & Honorary Consultant, Division of Population Medicine, School of Medicine, Cardiff University, Heath Park, Cardiff, CF14 4XN UK; 4 Department of Biochemistry, Alder Hey Children’s NHS FT, East Prescott Road, Liverpool, L122AP UK

## Erratum

The original publication [1] is missing one reference in the reference list. This reference should be also cited within the text. This error was caused during typesetting. The publisher apologizes to the readers for the inconvenience caused. The correct reference and citation can be found here:

13. Martin Bland J, Altman D. Statistical methods for assessing agreement between two methods of clinical measurement. The Lancet. 1986. 327(8476):307-10. doi:10.1016/S0140-6736(86)90837-8.

The Bland-Altman [13] analysis of agreement method was used to compare local and central measurements.
